# The Debate on Salicin and the Salicylates in Rheumatism

**Published:** 1882-03

**Authors:** 


### THE DEBATE ON SALICIN AND THE SALICYLATES IN RHEUMATISM.

The Medical Society of London may be congratulated upon the inauguration of a discussion which will probably have a great influence upon the therapeutics of rheumatic fever. The opening paper of Dr. Hilton Fagge is, by itself, a most important contribution to the subject, and, together with the communication of Dr. Isambard Owen, which followed, giving statistics from St. George's Hospital, have already brought a mass of evidence upon the influence exerted by salicin and its derivatives upon the disease in question. Several other statistics and analyses are to be laid before the Society, so that on the conclusion of the debate we may hope to be in possession of as large a body of facts concerning one particular line of treatment as have ever been presented, even in acute rheumatism—the favored field for such inquiry. A sufficient length of time has now elapsed since the introduction of these drugs into medicine to enable us to arrive at some definite conclusion concerning their action; and such a conclusion can only be arrived at impartially, freed from bias or prejudice, by carefully compiled statistics. Not that statistics, as Dr. Fagge justly remarked, can give the whole truth; they present us with only one aspect, and taken by themselves are liable to much misconstruction; but the statistical method, so far as it goes, and guarded by critical scrutiny of the individual facts, is of great value in medicine. Awaiting the completion of this debate, or rather "symposium," before examining it critically, we may briefly indicate the lines taken by the speakers. Dr. Fagge's remarks covered the whole question in an exhaustive manner; and many intending speakers will no doubt find that he has anticipated much that they would have said. For, in spite of the unfavorable conclusions which Dr. Greenhow felt compelled to draw from his elaborate and critical analyses of his series of cases communicated to the Clinical Society two years ago, there is certainly no evidence that these remedies are losing ground in medical favor. On the other hand, they are being employed more than ever. Dr. Fagge endeavored to show that Dr. Greenhow was not justified in drawing his conclusions, and, among other points, criticised his elimination of mild cases from his statistics; but we feel sure that if Dr. Greenhow erred at all it was on the side of caution, and his inferences were drawn after very close analysis of the cases before him. However, the large number of cases accumulated by Dr. Fagge leave little room for doubt as to the efficacy of the drug. The ground for debate will, we imagine, hardly be found here; few can question the potency of the remedy in cutting short the pyrexia and joint affection, although it does not appear to influence the cardiac manifestations. The open questions are the best mode of administration of the drug, the amount necessary to be given, the relation of relapse or "recrudescence" to the treatment, if any relation there be, and the toxic effects generally ascribed to it, especially those involving the heart and nervous system. These and other points will doubtless be dealt with, and we hope it may be possible for the Society to allot sufficient time for their discussion; for, although Dr. Fagge left no point untouched, the opportunity for collating a vast amount of experience is one which should not be lost. Dr. Isambard Owen's statistics, most elaborately worked out in relation to the severity of the cases treated, and in comparison with treatment by alkalies, were also in favor of the salicyl compounds; but many points were raised which we have not space to notice. Dr. Maclagan, the third and last speaker, concurred with Dr. Fagge in his criticism of Dr. Greenhow's conclusions, and entered at some length into his own special views concerning rheumatism being a malarial disease, and dependent upon the development of a virus which salicin and its derivatives are capable of destroying. Apart from his theoretical explanation of its *modus operandi*, it must not be forgotten that it is to Dr. Maclagan that we owe the employment in rheumatism of these drugs, which are now being put to the test of statistics. It remains to be seen whether they possess all the virtues that have been claimed for them.—*London Lancet.*

